# Clinical outcomes by serum potassium levels for patients hospitalized for heart failure: Secondary analysis of data from the China National Heart Failure Registry

**DOI:** 10.1002/clc.24114

**Published:** 2023-08-14

**Authors:** Jingmin Zhou, Xuejuan Jin, Jun Zhou, Yamei Xu, Xiaotong Cui, Michael Fu, Kai Hu, Junbo Ge, the CN‐HF Investigators

**Affiliations:** ^1^ Department of Cardiology, Shanghai Institute of Cardiovascular Disease, Zhongshan Hospital Fudan University Shanghai China; ^2^ Department of Epidemiology, Shanghai Institute of Cardiovascular Disease, Zhongshan Hospital Fudan University Shanghai China; ^3^ Section of Cardiology, Department of Medicine, Sahlgrenska University Hospital/Östra Hospital University of Gothenburg Gothenburg Sweden

**Keywords:** cohort study, heart failure, hyperkalemia, hypokalemia, prognosis, renin‐angiotensin‐aldosterone system inhibitor, serum potassium

## Abstract

**Background:**

Dyskalemia is a mortality risk factor in patients with heart failure (HF).

**Hypothesis:**

We described the prevalence of dyskalemia, and clinical outcomes by serum potassium (sK) levels, in Chinese patients hospitalized for HF.

**Methods:**

In this secondary analysis of the prospective China National Heart Failure Registry, adult patients hospitalized between January 1, 2013 and June 30, 2015 who had at least one baseline sK measurement were followed for up to 3 years after discharge. The use of renin–angiotensin–aldosterone system inhibitors at baseline and clinical outcomes during follow‐up were compared among sK groups.

**Results:**

Among 6950 patients, 5529 (79.6%) had normokalemia (sK >3.5–5.0 mmol/L), 1113 (16.0%) had hypokalemia (sK 0–3.5 mmol/L), and 308 (4.4%) had hyperkalemia (sK >5.0 mmol/L). Baseline characteristics that were most common in patients with hyperkalemia than those with hypo‐ and normokalemia included older age, HF with reduced ejection fraction, New York Heart Association Class III/IV status, hypertension, and chronic kidney disease. Use of angiotensin‐converting enzyme inhibitors (ACEIs) or angiotensin receptor blockers (ARBs) differed across sK groups (*p* = .0001); reported in 64.1%, 63.4%, and 54.5% of patients with hypo‐, normo‐, and hyperkalemia, respectively. Overall, 26.6%, 28.6%, and 36.0% of patients with hypo‐, normo‐, and hyperkalemia had rehospitalization for worsened HF, or cardiovascular mortality; *p* = .0057 for between‐group comparison.

**Conclusions:**

Patients with hyperkalemia received ACEIs or ARBs for HF treatment at baseline less frequently than those with hypo‐ or normokalemia, and had worse prognoses. This warrants further investigation into effective hyperkalemia management in HF.

## Graphical abstract

Among real‐world Chinese patients hospitalized for heart failure, those with hyperkalemia received renin–angiotensin–aldosterone system inhibitors (RAASi) at baseline less frequently than those with hypo‐ or normokalemia, and had worse prognoses. Long‐term hyperkalemia management may allow RAASi optimization and improve clinical outcomes in this group, and should be evaluated further.

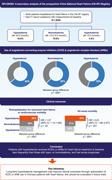

## 1 INTRODUCTION

Heart failure (HF) is a major and growing global health burden, being the end stage of many chronic diseases including cardiovascular (CV) diseases, hypertension, and diabetes.^1^, ^2^ In China, over 12 million people were estimated to be living with HF in 2017, with an age‐standardized prevalence rate of 1.10%.^3^ The burden of HF in China is expected to rise, with an aging population and an increase in chronic comorbidities as risk factors.^2^, ^4^

The prognosis for Chinese patients hospitalized for HF remains poor, with high reported rates of rehospitalisation and mortality.^3^, ^4^ In‐hospital or 3‐day postdischarge mortality rate in patients hospitalized for HF reached 3.2%, and more than 20% either died or were rehospitalized within a median of approximately 1‐month postdischarge.^4^ Over 40% of patients hospitalized for HF had at least three hospitalization episodes.^3^ This warrants strategies to improve the management of HF.

In patients with HF, dyskalemia (hypokalemia or hyperkalemia) is a common complication, arising due to pathophysiologic changes underlying HF itself, related comorbidities and medications used.^5^ Dyskalemia increases the risk of potentially fatal cardiac arrhythmias.^6^ Hyperkalemia may also dictate dose reduction or discontinuation of HF treatments, resulting in poor prognoses.^5^, ^7^ While current Chinese guidelines recommend renin–angiotensin–aldosterone system inhibitors (RAASis) to treat HF, RAASi treatment is reportedly underutilized in China (received by 23%–48% of patients).^8^, ^9^

Among Western patients with HF, both hypokalemia and hyperkalemia are associated with increased mortality risk compared with normokalemia, showing a U‐shaped relationship.^10^, ^11^, ^12^, ^13^, ^14^, ^15^, ^16^ Patients with hyperkalemia are also more likely to discontinue RAASi treatment than others.^11^ To our knowledge, such data among Chinese patients with HF are lacking.

The China National Heart Failure (CN‐HF) Registry (ClinicalTrials.gov, NCT02079428) was a nationwide, multicenter, prospective cohort study that aimed to understand the clinical characteristics, treatment, and prognosis of Chinese patients hospitalized for HF.^17^ Here, in the SPLENDID study, we described the baseline characteristics, including serum potassium (sK) levels and RAASi use, and subsequent clinical outcomes in Chinese patients hospitalized for HF, across all ejection fractions, enrolled in the CN‐HF Registry. We aimed to inform the risk of dyskalemia and the importance of improving sK management in clinical practice.

## 2 METHODS

### 2.1 Study design and patients

The CN‐HF Registry enrolled adult patients hospitalized for HF between January 1, 2013 and June 30, 2015 from 45 hospitals across 20 provinces in China. Using a two‐stage sampling method, candidate hospitals were first stratified by geographical regions and tiers, followed by randomized sampling according to the total population sizes in each stratum. Eligible study centers were second‐ and third‐tier hospitals with cardiology and/or geriatrics departments that were representative of their respective geographical areas; had accessible medical records; had qualified medical staff performing accurate and reliable data extraction; and had passed the qualification review by relevant academic committees. Eligible patients were those from the selected hospitals who received an HF diagnosis, upon discharge from the cardiology or cardiovascular department or upon death during hospitalization. SPLENDID study was a secondary analysis of the CN‐HF Registry, whereby patients who had at least one sK measurement during baseline hospitalization were included.

SPLENDID was conducted in accordance with the Declaration of Helsinki, Guidelines for Good Clinical Practice, and the applicable legislation on retrospective cohort studies. All patients in the CN‐HF cohort provided written informed consent before enrollment. The final protocol was approved by the Institutional Review Board and the Independent Ethics Committees of Zhongshan Hospital Fudan University and each participating hospital. The manuscript was developed in alignment with the Strengthening the Reporting of Observational Studies in Epidemiology (STROBE) reporting guidelines.

### 2.2 Procedures

Baseline characteristics (at first hospital admission) that were extracted from the registry included sK measurements (first available value), medical history, HF classification based on left ventricular ejection fraction (LVEF) from the first echocardiography, and RAASi use. Patients were grouped according to sK levels: hypokalemia (0–3.5 mmol/L), normokalemia (>3.5–5.0 mmol/L), and hyperkalemia (>5.0 mmol/L). HF was categorized as having preserved ejection fraction (HFpEF; LVEF ≥ 50%), mid‐range ejection fraction (HFmEF; LVEF 40%–49%), or reduced ejection fraction (HFrEF; LVEF < 40%). Prespecified RAASis included angiotensin‐converting enzyme inhibitors (ACEIs), angiotensin receptor blockers (ARBs), and mineralocorticoid receptor antagonists (MRAs); there were no data on angiotensin receptor neprilysin inhibitors in the registry. Sodium‐glucose cotransporter 2 inhibitors were not available at study enrollment.

Patients were followed up for clinical endpoints through outpatient visits or telephone calls every 3–6 months for up to 3 years after discharge or until death, whichever occurred earlier. The dates of occurrence (if any) of rehospitalization for worsened HF, CV mortality, and all‐cause mortality for each patient were extracted from the registry.

### 2.3 Endpoints

The primary endpoint was the proportion of patients in each sK category who experienced a composite of rehospitalization for worsened HF, or CV mortality, during follow‐up. Secondary endpoints included the proportions of patients in each sK category who experienced the individual components of the primary endpoint, and all‐cause mortality, during follow‐up. Other secondary endpoints were the distribution of baseline sK levels; the proportions of patients with sK levels >5.0 mmol/L and >5.5 mmol/L at baseline; and the proportions of patients in each sK category who received RAASi at baseline.

### 2.4 Statistical analysis

Among 7171 adult patients hospitalized for HF enrolled in the CN‐HF Registry, 6950 patients had at least one sK measurement during baseline hospitalization, and were included in the full analysis set (FAS) of SPLENDID (Figure S1). Based on an estimated rate of hyperkalemia of 4.8% (i.e., approximately 333 patients in the FAS) at baseline, and assuming a primary endpoint event rate of 27.5% for this group, a precision level of 4.8% would be achieved. Assuming 251 patients (3.6%) had hypokalemia and 6366 (91.6%) had normokalemia with respective primary endpoint event rates of 26.5% and 23.0%, the precision levels achieved would be 5.5% and 1.0% for the corresponding groups.^13^

Statistical analyses were performed using SAS, version 9.3 (SAS Institute Inc.). All data were categorical, shown as frequencies and percentages (with 95% confidence intervals for clinical outcomes), and based on non‐missing data unless otherwise specified. Statistical comparisons of baseline characteristics and clinical outcomes among sK categories were conducted post hoc. Differences in baseline characteristics were analyzed using the Pearson's Chi‐square test (for binary variables), the Cochran–Mantel–Haenszel test (for variables with multiple groups), or the Cochran Armitage trend test (for RAASi use). Differences in the rates of clinical events were analyzed using the Cochran–Mantel–Haenszel test (row mean scores differ). The association of hyperkalemia (vs. hypo‐ and normokalemia) with clinical outcomes was evaluated using logistic regression analysis in the FAS and by HF type.

## 3 RESULTS

### 3.1 Baseline sK and other characteristics

Among 6950 patients in the FAS, 5529 (79.6%) had normokalemia, 1113 (16.0%) had hypokalemia, and 308 (4.4%) had hyperkalemia at baseline (Table 1). Overall, 96 patients (1.4%) had sK >5.5 mmol/L. Specifically, the proportions of patients with sK >5.0–5.5, >5.5–6.0, >6.0–6.5, >6.5–7.0, and >7.0 mmol/L were 3.1%, 0.7%, 0.4%, 0.1%, and 0.1%, respectively.

**Table 1:** Distribution of patients across baseline serum potassium categories.

Baseline sK (mmol/L)	FAS (*N* = 6950)
Hypokalemia	
≤3.5	1113 (16.0)
Normokalemia	
>3.5–5.0	5529 (79.6)
Hyperkalemia	
>5.0	308 (4.4)
>5.0–5.5	212 (3.1)
>5.5	96 (1.4)
>5.5–6.0	52 (0.7)
>6.0–6.5	27 (0.4)
>6.5–7.0	9 (0.1)
>7.0	8 (0.1)

*Note*: Data presented as *n* (%).

Abbreviations: FAS, full analysis set; sK, serum potassium.

Mean age was 69.6 ± 13.6 years (94.9% of patients were ≥45 years and 43.3% were ≥75 years), and 59.3% were male (Table 2). Overall, 4716 patients (67.9%) had hypertension, 2158 (31.1%) had diabetes mellitus, and 766 (11.0%) had chronic kidney disease (CKD); 3848 patients (55.4%) were classified as New York Heart Association (NYHA) Class III/IV. Most patients (2865 [41.2%]) had HFpEF, followed by 1949 (28.0%) with HFrEF and 663 (9.5%) with HFmEF. Several baseline characteristics differed across sK groups, including age (*p* < .05), NYHA Class III/IV status (*p* < .0001), HF type (*p* < .01), and comorbidities (hypertension [*p* < .01] and CKD [*p* < .0001]). For patients with hyperkalemia compared with hypo‐ and normokalemia, respectively, we observed a higher proportion of patients with age ≥75 years (53.2%, 42.6%, and 42.8%), NYHA Class III/IV HF (72.1%, 57.4%, and 54.0%), HFrEF (31.2%, 23.8%, and 28.7%), hypertension (75.0%, 69.4%, and 67.2%) and CKD (37.0%, 7.9%, and 10.2%).

**Table 2:** Baseline characteristics of patients grouped according to serum potassium levels.

Characteristic	FAS (*N* = 6950)	sK categories			*p* value^a^
		Hypokalemia (*n* = 1113)	Normokalemia (*n* = 5529)	Hyperkalemia (*n* = 308)	
**Demographics**					
Age (years)					<.05^b^
18–44	357 (5.1)	55 (4.9)	293 (5.3)	9 (2.9)	
45–64	1816 (26.1)	310 (27.9)	1445 (26.1)	61 (19.8)	
65–74	1770 (25.5)	274 (24.6)	1422 (25.7)	74 (24.0)	
≥75	3007 (43.3)	474 (42.6)	2369 (42.8)	164 (53.2)	
Sex					<.001
Male	4119 (59.3)	604 (54.3)	3338 (60.4)	177 (57.5)	
**HF and concomitant diseases**					
NYHA Class III/IV	3848 (55.4)	639 (57.4)	2987 (54.0)	222 (72.1)	<.0001
HF type					<.01^c^
HFrEF	1949 (28.0)	265 (23.8)	1588 (28.7)	96 (31.2)	
HFmEF	663 (9.5)	114 (10.2)	517 (9.4)	32 (10.4)	
HFpEF	2865 (41.2)	474 (42.6)	2293 (41.5)	98 (31.8)	
Unknown (missing baseline LVEF)	1473 (21.2)	260 (23.4)	1131 (20.5)	82 (26.6)	
Hypertension	4716 (67.9)	772 (69.4)	3713 (67.2)	231 (75.0)	<.01
Diabetes mellitus	2158 (31.1)	351 (31.5)	1696 (30.7)	111 (36.0)	>.05
Chronic kidney disease	766 (11.0)	88 (7.9)	564 (10.2)	114 (37.0)	<.0001

*Note*: Data presented as *n* (%) unless otherwise specified.

Abbreviations: FAS, full analysis set; HF, heart failure; HFmEF, HF with mid‐range ejection fraction; HFpEF, HF with preserved ejection fraction; HFrEF, HF with reduced ejection fraction; LVEF, left ventricular ejection fraction; NYHA, New York Heart Association; sK, serum potassium.

^a^ Calculated using Chi‐square test unless otherwise specified.

^b^ Calculated using Cochran–Mantel–Haenszel test (nonzero correlation).

^c^ Calculated using Cochran–Mantel–Haenszel test (row mean scores differ).

### 3.2 Baseline RAASi use by sK categories

Overall, 5552 of 6950 patients (79.9%) were treated with RAASis (ACEIs, ARBs, or MRAs) at baseline, with 4388 patients (63.1%) receiving ACEIs or ARBs and 4359 (62.7%) receiving MRAs (Table 3). When grouped by sK categories, 81.0%, 79.7%, and 78.2% of patients in the hypo‐, normo‐, and hyperkalemia groups, respectively, received any RAASi (*p* = .0087). Use of ACEIs and ARBs differed across sK groups (*p* = .0001), while MRA use was similar across sK groups (*p* = .1927). Rate of ACEI or ARB use was lower among patients with hyperkalemia (54.5%) than those with hypo‐ or normokalemia (64.1% and 63.4%, respectively).

**Table 3:** Baseline RAASi treatment rates by serum potassium categories.

Treatment for HF	FAS (*N* = 6950)	Hypokalemia (*n* = 1113)	Normokalemia (*n* = 5529)	Hyperkalemia (*n* = 308)	*p* value^a^
RAASi^b^	5552 (79.9)	902 (81.0)	4409 (79.7)	241 (78.2)	.0087
ACEI or ARB	4388 (63.1)	713 (64.1)	3507 (63.4)	168 (54.5)	.0001
MRA	4359 (62.7)	718 (64.5)	3439 (62.2)	202 (65.6)	.1927

*Note*: Data presented as *n* (%) unless otherwise specified.

Abbreviations: ACEI, angiotensin‐converting enzyme inhibitor; ARB, angiotensin receptor blocker; FAS, full analysis set; MRA, mineralocorticoid receptor antagonist; RAASi, renin–angiotensin‐aldosterone system inhibitor.

^a^ Two‐tailed Cochran Armitage trend test. *Z* = 2.6231 for RAASi; *Z* = 3.8318 for ACEI or ARB; *Z* = 1.3026 for MRA.

^b^ RAASis included ACEIs, ARBs, or MRAs.

### 3.3 Clinical outcomes by sK categories

During follow‐up, 1990 of 6950 patients (28.6%) experienced rehospitalization for worsened HF, or CV mortality, with event rates that differed across sK groups (*p* = .0057) (Table 4). A higher proportion of patients in the hyperkalemia group (36.0%) experienced this primary composite endpoint than those in the hypo‐ (26.6%) or normokalemia (28.6%) groups. Overall, the individual components of the primary composite endpoint, namely rehospitalization for worsened HF and CV mortality, occurred in 1922 (27.7%) and 805 (11.6%) patients, respectively; their rates differed across sK groups (*p* = .0062 for rehospitalization for worsened HF; *p* = .0002 for CV mortality) and showed similar trends to the composite endpoint. All‐cause mortality, which occurred in 1494 patients (21.5%) in the FAS, also differed across sK groups (*p* < .0001), being observed in 41.6%, 17.8%, and 21.1% of patients with hyper‐, hypo‐ and normokalemia, respectively.

**Table 4:** Clinical endpoint event rates by baseline serum potassium categories.

Endpoint events	FAS (*N* = 6950)	sK categories			*p* value^a^
		Hypokalemia (*n* = 1113)	Normokalemia (*n* = 5529)	Hyperkalemia (*n* = 308)	
Rehospitalization for worsened HF, or CV mortality	1990 (28.6 [27.6–29.7])	296 (26.6 [24.0–29.3])	1583 (28.6 [27.4–29.8])	111 (36.0 [30.7–41.7])	.0057
Rehospitalization for worsened HF	1922 (27.7 [26.6–28.7])	284 (25.5 [23.0–28.2])	1532 (27.7 [26.5–28.9])	106 (34.4 [29.1–40.0])	.0062
CV mortality	805 (11.6 [10.8–12.4])	108 (9.7 [8.0–11.6])	639 (11.6 [10.7–12.4])	58 (18.8 [14.6–23.7])	.0002
All‐cause mortality	1494 (21.5 [20.5–22.5])	198 (17.8 [15.6–20.2])	1168 (21.1 [20.1–22.2])	128 (41.6 [36.0–47.3])	<.0001

*Note*: Data presented as *n* (% [95% CI]) unless otherwise specified.

Abbreviations: CI, confidence interval; CV, cardiovascular; FAS, full analysis set; HF, heart failure.

^a^ Calculated using Cochran–Mantel–Haenszel test (row mean scores differ).

In the FAS, hyperkalemia was associated with a statistically significant 37% increase in risk for the composite of rehospitalization for worsened HF, or CV mortality, compared with hypo‐ and normokalemia (Table 5). To account for the potential effect of different HF types on prognoses, the association of hyperkalemia with clinical outcomes was separately assessed in the HFrEF, HFmEF, and HFpEF subgroups. There was a trend of increased risk of the primary endpoint in patients with hyperkalemia versus those with hypo‐ or normokalemia across all HF types, although this did not reach statistical significance in the HFrEF subgroup. Similar trends of increased risk of the individual components of the primary endpoint with hyperkalemia were observed; these were statistically significant across all HF types except for rehospitalization for worsened HF in the HFrEF subgroup. The risk for all‐cause mortality increased by approximately two‐fold for hyperkalemia versus hypo‐ and normokalemia (*p* < .001) in the FAS and in all subgroups defined by HF type.

**Table 5:** Association of clinical endpoint events with hyperkalemia (sK >5.0 mmol/L) versus normo‐ and hypokalemia (sK ≤5.0 mmol/L) analyzed by heart failure type.

	FAS		HFrEF		HFmEF		HFpEF	
sK	≤5.0 (*n* = 6642)	>5.0 (*n* = 308)	≤5.0 (*n* = 1853)	>5.0 (*n* = 96)	≤5.0 (*n* = 631)	>5.0 (*n* = 32)	≤5.0 (*n* = 2767)	>5.0 (*n* = 98)
Rehospitalization for worsened HF, or CV mortality								
Events, *n* (%)	1879 (28.3)	111 (36.0)	984 (30.3)	57 (32.0)	895 (26.3)	54 (41.5)	698 (25.2)	36 (36.7)
HR (95% CI)^a^	1.37 (1.13–1.66)^b^		1.22 (0.93–1.59)		1.53 (1.16–2.01)^b^		1.45 (1.04–2.02)^b^	
Rehospitalization for worsened HF								
Events, *n* (%)	1816 (27.3)	106 (34.4)	938 (28.9)	53 (29.8)	878 (25.8)	53 (40.8)	686 (24.8)	35 (35.7)
HR (95% CI)^a^	1.47 (1.21–1.78)^b^		1.26 (0.96–1.67)		1.70 (1.29–2.25)^b^		1.57 (1.12–2.20)^b^	
CV mortality								
Events, *n* (%)	747 (11.3)	58 (18.8)	439 (13.5)	32 (18.0)	308 (9.1)	26 (20.0)	225 (8.1)	18 (18.4)
HR (95% CI)^a^	1.84 (1.41–2.41)^c^		1.55 (1.09–2.23)^b^		2.22 (1.49–3.32)^c^		2.28 (1.41–3.69)^b^	
All‐cause mortality								
Events, *n* (%)	1366 (20.6)	128 (41.6)	783 (24.1)	85 (47.8)	583 (17.2)	43 (33.1)	447 (16.2)	32 (32.7)
HR (95% CI)^a^	2.23 (1.86–2.67)^c^		2.31 (1.84–2.88)^c^		1.95 (1.43–2.66)^c^		2.05 (1.43–2.94)^c^	

Abbreviations: CI, confidence interval; CV, cardiovascular; FAS, full analysis set; HFmEF, HF with mid‐range ejection fraction; HFpEF, HF with preserved ejection fraction; HFrEF, HF with reduced ejection fraction; HR, hazard ratio; LVEF, left ventricular ejection fraction; sK, serum potassium.

^a^ Calculated using logistic regression analysis; based on unadjusted models. Reference group: sK >5.0 mmol/L.

b *p* < .05.

^c^ *p* < .001.

## 4 DISCUSSION

Using data from the prospective real‐world CN‐HF Registry, we analyzed, by sK levels, the baseline characteristics and clinical outcomes of Chinese patients hospitalized for HF. Dyskalemia is common in patients with HF, with reported frequencies of 3.0%–25.8% for hypokalemia and 5.7%–39.0% for hyperkalemia.^11^, ^14^, ^15^, ^16^, ^18^, ^19^ Consistent with previous reports, potential risk factors for hyperkalemia in patients with HF included older age, HF comorbidities, and worse NYHA class function.^14^, ^15^, ^16^, ^20^, ^21^, ^22^, ^23^, ^24^, ^25^, ^26^ Among HF subtypes, hyperkalemia versus hypo‐ and normokalemia was observed more often in patients with HFrEF and less often in those with HFpEF. This may be due to greater use of RAASi, which can potentially cause hyperkalemia, among patients with HFrEF as recommended by clinical guidelines,^27^ although the use of RAASi by sK levels was not analyzed based on HF type in the present study. Patients with hypokalemia appeared to have comparable baseline characteristics to those with normokalemia in this cohort, unlike in other studies.^13^, ^15^, ^16^, ^28^ The identification of baseline characteristics associated with sK level may be useful for dyskalemia risk prediction in patients with HF.

As RAASi can induce hyperkalemia, its real‐world utilization for HF treatment remains suboptimal.^5^, ^29^, ^30^, ^31^, ^32^ Our findings indicated that ACEI or ARB use at baseline were lower in patients with hyperkalemia than those with hypo‐ or normokalemia. MRA use was not significantly different across sK categories, which may be due to the clinical practice in China where, rather than MRA, the use of ACEI or ARB is usually adjusted in patients upon the development of hyperkalemia. Real‐world database studies in Western patients with HF who received RAASi have shown a direct association of hyperkalemia with treatment dose reduction or discontinuation.^30^, ^31^ We observed a J‐shaped relationship with hyperkalemia being associated with adverse clinical outcomes. Both hyperkalemia and hypokalemia were previously associated with increased risk for CV events and all‐cause mortality in Western populations, suggesting a U‐shaped relationship.^10^, ^11^, ^12^, ^13^, ^14^, ^15^, ^16^ Differences between these studies may be due to inter‐ethnic differences, as well as variations in clinical practice between countries.^33^, ^34^, ^35^, ^36^ The lack of an association between adverse clinical outcomes and hypokalemia in our study might be due to the easy correction of hypokalemia in the clinic, since subsequent normalization of sK was not considered. Despite differences in prognoses among HF types,^37^, ^38^ we consistently observed a higher risk for adverse clinical outcomes among patients with hyperkalemia across all HF subgroups. While we did not assess the association between RAASi use and clinical outcomes in this study, our data support real‐world Western studies where patients who discontinued RAASis, or those on reduced doses, due to hyperkalemia, exhibited increased incidence of major adverse cardiac events and mortality than those on maximum dose.^30^, ^31^

Collectively, findings from SPLENDID and previous studies support the importance of long‐term hyperkalemia management in HF, including in Chinese patients, which can potentially improve clinical outcomes by allowing optimal use of RAASis.^30^, ^31^, ^39^ Current guidelines from the European Society of Cardiology suggest the use of novel potassium binders (sodium zirconium cyclosilicate [SZC] and patiromer) for the management of RAASi‐associated hyperkalemia.^32^ According to a phase 3 study, correction of hyperkalemia and maintenance of normokalemia were observed with SZC treatment for up to 12 months among outpatients, without the need for RAASi medication restrictions; 87% of the study participants were able to maintain or increase their baseline RAASi treatment dosages.^40^ Further randomized controlled trials that directly evaluate the benefits of hyperkalemia management on RAASi optimization and clinical outcomes in Chinese patients with HF are warranted.

A strength of this study is the use of data from a prospective real‐world registry, which included patients across all ranges of HF ejection fraction. A key limitation is the retrospective nature of the analysis. Several variables, including patients’ diet, medications (e.g., diuretics), potassium supplementation, and the causes of hyperkalemia, were not available to adjust for confounding. Thus, the association between sK levels and clinical outcomes should be interpreted cautiously. Second, clinical outcomes were analyzed only based on sK at baseline, negating the effect of subsequent sK fluctuations. It was previously shown that persistent dyskalemia was linked to a higher mortality risk versus sK normalization, hence longitudinal monitoring of dynamic sK changes would provide a more accurate prediction of clinical outcomes.^13^

## 5 CONCLUSIONS

In this secondary analysis of a real‐world registry, dyskalemia was observed in more than 20% of Chinese patients hospitalized for HF. Several baseline characteristics, including older age, worse NYHA class function, HFrEF, hypertension, and CKD, were most common in patients with hyperkalemia. Compared with the hypo‐ and normokalemia groups, a lower proportion of patients with hyperkalemia were prescribed ACEIs or ARBs for the treatment of HF at baseline, but a higher proportion experienced rehospitalization for worsened HF, CV mortality, and all‐cause mortality after discharge regardless of HF type. The effective management of hyperkalemia in patients with HF should be evaluated for its potential to allow optimal use of RAASi treatment and to improve clinical outcomes.

## AUTHOR CONTRIBUTIONS

**Jingmin Zhou, Michael Fu, Kai Hu, and Junbo Ge** conceived and designed the study. **Jingmin Zhou, Xuejuan Jin, Jun Zhou, Yamei Xu, and Xiaotong Cui** acquired, analyzed, or interpreted the data. **Xuejuan Jin and Jun Zhou** conducted the statistical analysis. **Jingmin Zhou and Xuejuan Jin** drafted the manuscript. **Michael Fu and Junbo Ge** critically revized the manuscript for important intellectual content. **Jingmin Zhou** obtained funding for the study. **Jingmin Zhou and Junbo Ge** provided administrative, technical, or material support. **Jingmin Zhou, Michael Fu, and Junbo Ge** provided supervision. All authors have read and approved the final manuscript.

## CONFLICT OF INTEREST STATEMENT

The authors declare no conflicts of interest.

## Supporting information

Supporting information.

## Data Availability

The data that support the findings of this study are available from the corresponding author upon reasonable request.

## References

[1] Ponikowski P , Anker SD , AlHabib KF , et al. Heart failure: preventing disease and death worldwide. ESC Heart Failure. 2014;1:4‐25. 28834669 10.1002/ehf2.12005

[2] Savarese G , Lund LH . Global public health burden of heart failure. Card Fail Rev. 2017;3:7‐11. 28785469 10.15420/cfr.2016:25:2 PMC5494150

[3] Wang H , Chai K , Du M , et al. Prevalence and incidence of heart failure among urban patients in China: a national population‐based analysis. Circulation: Heart Failure. 2021;14:e008406. 34455858 10.1161/CIRCHEARTFAILURE.121.008406

[4] Li L , Liu R , Jiang C , et al. Assessing the evidence‐practice gap for heart failure in China: the Heart Failure Registry of Patient Outcomes (HERO) study design and baseline characteristics. Eur J Heart Fail. 2020;22:646‐660. 31820513 10.1002/ejhf.1630

[5] Ferreira JP , Butler J , Rossignol P , et al. Abnormalities of potassium in heart failure: JACC state‐of‐the‐art review. JACC. 2020;75:2836‐2850. 32498812 10.1016/j.jacc.2020.04.021

[6] Weiss JN , Qu Z , Shivkumar K . Electrophysiology of hypokalemia and hyperkalemia. Circ Arrhythm Electrophysio. 2017;10:e004667. 10.1161/CIRCEP.116.004667 PMC5399982 28314851

[7] Rossignol P , Lainscak M , Crespo‐Leiro MG , et al. Unravelling the interplay between hyperkalaemia, renin‐angiotensin‐aldosterone inhibitor use and clinical outcomes. Data from 9222 chronic heart failure patients of the ESC‐HFA‐EORP Heart Failure Long‐Term Registry. Eur J Heart Fail. 2020;22:1378‐1389. 32243669 10.1002/ejhf.1793

[8] Heart Failure Group of Chinese Society of Cardiology of Chinese Medical Association, Chinese Heart Failure Association of Chinese Medical Doctor Association, Editorial Board of Chinese Journal of Cardiology . [Chinese guidelines for the diagnosis and treatment of heart failure 2018]. Zhonghua Xin Xue Guan Bing Za Zhi. 2018;46:760‐789. 30369168 10.3760/cma.j.issn.0253-3758.2018.10.004

[9] Huang J , Yin H , Zhang M , Ni Q , Xuan J . Understanding the economic burden of heart failure in China: impact on disease management and resource utilization. J Med Econ. 2017;20:549‐553. 28286996 10.1080/13696998.2017.1297309

[10] Aldahl M , Jensen ASC , Davidsen L , et al. Associations of serum potassium levels with mortality in chronic heart failure patients. Eur Heart J. 2017;38:2890‐2896. 29019614 10.1093/eurheartj/ehx460

[11] Linde C , Qin L , Bakhai A , et al. Serum potassium and clinical outcomes in heart failure patients: results of risk calculations in 21 334 patients in the UK. ESC Heart Failure. 2019;6:280‐290. 30629342 10.1002/ehf2.12402 PMC6437434

[12] McEwan P , Hurst M , Hoskin L , et al. The relationship between duration of heart failure, serum potassium concentration and adverse clinical outcomes. Eur Heart J. 2020;41:ehaa946.0943.

[13] Núñez J , Bayés‐Genís A , Zannad F , et al. Long‐term potassium monitoring and dynamics in heart failure and risk of mortality. Circulation. 2018;137:1320‐1330. 29025765 10.1161/CIRCULATIONAHA.117.030576

[14] Thomsen RW , Nicolaisen SK , Hasvold P , et al. Elevated potassium levels in patients with congestive heart failure: occurrence, risk factors, and clinical outcomes. J Am Heart Assoc. 2018;7:e008912. 29789332 10.1161/JAHA.118.008912 PMC6015368

[15] Matsushita K , Sang Y , Yang C , et al. Dyskalemia, its patterns, and prognosis among patients with incident heart failure: A nationwide study of US veterans. PLoS One. 2019;14:e0219899. 31393910 10.1371/journal.pone.0219899 PMC6687136

[16] Savarese G , Xu H , Trevisan M , et al. Incidence, predictors, and outcome associations of dyskalemia in heart failure with preserved, mid‐range, and reduced ejection fraction. JACC: Heart Failure. 2019;7:65‐76. 30553905 10.1016/j.jchf.2018.10.003

[17] Jin X , Cao J , Zhou J , et al. Outcomes of patients with anemia and renal dysfunction in hospitalized heart failure with preserved ejection fraction (from the CN‐HF Registry). IJ Heart & Vasculature. 2019;25:100415. 10.1016/j.ijcha.2019.100415 PMC6726881 31508483

[18] Collins AJ , Pitt B , Reaven N , et al. Association of serum potassium with all‐cause mortality in patients with and without heart failure, chronic kidney disease, and/or diabetes. Am J Nephrol. 2017;46:213‐221. 28866674 10.1159/000479802 PMC5637309

[19] Tromp J , Ter Maaten JM , Damman K , et al. Serum potassium levels and outcome in acute heart failure (data from the PROTECT and COACH trials). Am J Cardiol. 2017;119:290‐296. 27823598 10.1016/j.amjcard.2016.09.038

[20] Di Lullo L , Ronco C , Granata A , et al. Chronic hyperkalemia in cardiorenal patients: risk factors, diagnosis, and new treatment options. Cardiorenal Med. 2019;9:8‐21. 30359977 10.1159/000493395

[21] Musso C , Liakopoulos V , De Miguel R , Imperiali N , Algranati L . Transtubular potassium concentration gradient: comparison between healthy old people and chronic renal failure patients. Int Urol Nephrol. 2006;38:387‐390. 16868716 10.1007/s11255-006-0059-5

[22] Musso CG , Miguel R , Algranati L , Farias EDR . Renal potassium excretion: comparison between chronic renal disease patients and old people. Int Urol Nephrol. 2005;37:167‐170. 16132781 10.1007/s11255-004-2361-4

[23] Fudim M , Grodin JL , Mentz RJ . Hyperkalemia in heart failure: probably not O“K”. J Am Heart Assoc. 2018;7:e009429. 29789333 10.1161/JAHA.118.009429 PMC6015362

[24] Muzzarelli S , Maeder MT , Toggweiler S , et al. Frequency and predictors of hyperkalemia in patients ≥60 years of age with heart failure undergoing intense medical therapy. Am J Cardiol. 2012;109:693‐698. 22152974 10.1016/j.amjcard.2011.10.027

[25] Rakugi H , Yamakawa S , Sugimoto K . Management of hyperkalemia during treatment with mineralocorticoid receptor blockers: findings from esaxerenone. Hypertension Res. 2021;44:371‐385. 10.1038/s41440-020-00569-y PMC8019656 33214722

[26] Vardeny O , Claggett B , Anand I , et al. Incidence, predictors, and outcomes related to hypo‐ and hyperkalemia in patients with severe heart failure treated with a mineralocorticoid receptor antagonist. Circulation: Heart Failure. 2014;7:573‐579. 24812304 10.1161/CIRCHEARTFAILURE.114.001104

[27] Heidenreich PA , Bozkurt B , Aguilar D , et al. AHA/ACC/HFSA guideline for the management of heart failure: a report of the American College of Cardiology/American Heart Association Joint Committee on clinical practice guidelines. Circulation. 2022;145:e895‐e1032. 35363499 10.1161/CIR.0000000000001063

[28] Nilsson E , Gasparini A , Ärnlöv J , et al. Incidence and determinants of hyperkalemia and hypokalemia in a large healthcare system. Int J Cardiol. 2017;245:277‐284. 28735756 10.1016/j.ijcard.2017.07.035

[29] Chapman A , Gunning S . Real‐world associations between renin‐angiotensin‐aldosterone system inhibition therapy, hyperkalemia, and outcomes: a clinical and scientific call to action. J Am Heart Assoc. 2019;8:e014845. 31711386 10.1161/JAHA.119.014845 PMC6915277

[30] Linde C , Bakhai A , Furuland H , et al. Real‐world associations of renin‐angiotensin‐aldosterone system inhibitor dose, hyperkalemia, and adverse clinical outcomes in a cohort of patients with new‐onset chronic kidney disease or heart failure in the United Kingdom. J Am Heart Assoc. 2019;8:e012655. 31711387 10.1161/JAHA.119.012655 PMC6915283

[31] Epstein M , Reaven NL , Funk SE , McGaughey KJ , Oestreicher N , Knispel J . Evaluation of the treatment gap between clinical guidelines and the utilization of renin‐angiotensin‐aldosterone system inhibitors. Am J Manag Care. 2015;21:S212‐S220. 26619183

[32] Silva‐Cardoso J , Brito D , Frazão JM , et al. Management of RAASi‐associated hyperkalemia in patients with cardiovascular disease. Heart Fail Rev. 2021;26:891‐896. 33599908 10.1007/s10741-020-10069-3 PMC8149346

[33] Chen Y , Sang Y , Ballew SH , et al. Race, serum potassium, and associations with ESRD and mortality. Am J Kidney Dis. 2017;70:244‐251. 28363732 10.1053/j.ajkd.2017.01.044 PMC5526716

[34] Eriguchi R , Obi Y , Soohoo M , et al. Racial and ethnic differences in mortality associated with serum potassium in incident peritoneal dialysis patients. Am J Nephrol. 2019;50:361‐369. 31522173 10.1159/000502998 PMC6856395

[35] Hayes J , Kalantar‐Zadeh K , Lu JL , Turban S , Anderson JE , Kovesdy CP . Association of hypo‐ and hyperkalemia with disease progression and mortality in males with chronic kidney disease: the role of race. Nephron Clin Pract. 2012;120:c8‐c16. 22156587 10.1159/000329511 PMC3267990

[36] Kim T , Rhee CM , Streja E , et al. Racial and ethnic differences in mortality associated with serum potassium in a large hemodialysis cohort. Am J Nephrol. 2017;45:509‐521. 28528336 10.1159/000475997 PMC5546877

[37] Curtis JP , Sokol SI , Wang Y , et al. The association of left ventricular ejection fraction, mortality, and cause of death in stable outpatients with heart failure. JACC. 2003;42:736‐742. 12932612 10.1016/s0735-1097(03)00789-7

[38] Liang M , Bian B , Yang Q . Characteristics and long‐term prognosis of patients with reduced, mid‐range, and preserved ejection fraction: a systemic review and meta‐analysis. Clin Cardiol. 2022;45:5‐17. 35043472 10.1002/clc.23754 PMC8799045

[39] Cooper LB , Mentz RJ . Potassium abnormalities across the spectrum of heart failure. JACC: Heart Failure. 2019;7:77‐79. 30553906 10.1016/j.jchf.2018.11.001

[40] Spinowitz BS , Fishbane S , Pergola PE , et al. Sodium zirconium cyclosilicate among individuals with hyperkalemia: a 12‐month phase 3 study. Clin J Am Soc Nephrol. 2019;14:798‐809. 31110051 10.2215/CJN.12651018 PMC6556727

