# Supplementary material for: Clinical outcomes by serum potassium levels for patients hospitalized for heart failure: Secondary analysis of data from the China National Heart Failure Registry
Source: Clin Cardiol. 2023 Aug 14;46(11):1345–52. doi: 10.1002/clc.24114 (PMC10642319; doi:10.1002/clc.24114)
Supplement: Supplementary file 1 — Supporting information. [file CLC-46-1345-s001.docx]

**Clinical outcomes by serum potassium levels for patients hospitalized for heart failure: Secondary analysis of data from the China National Heart Failure Registry**


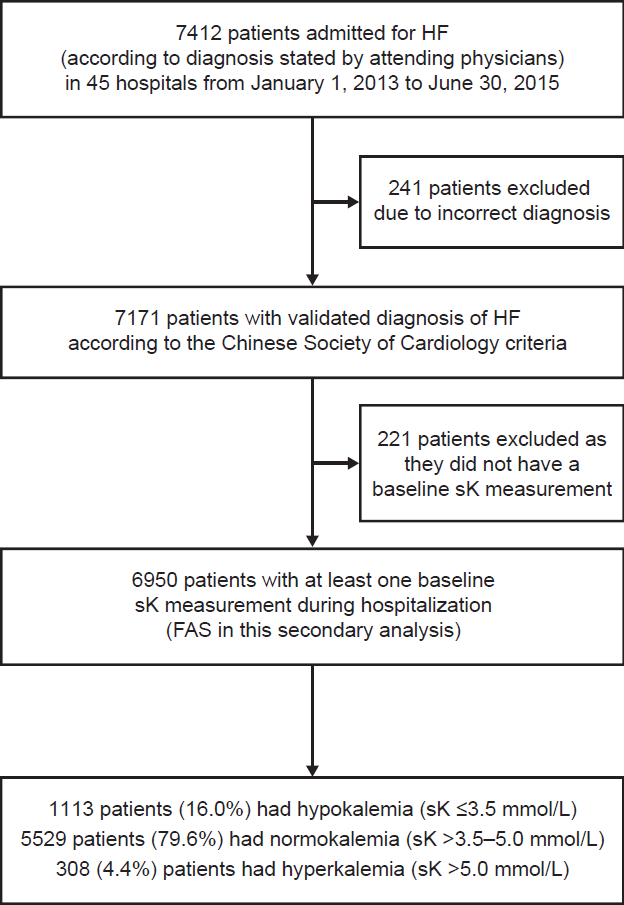


**FIGURE S1** SPLENDID study patient flow diagram.

FAS, full analysis set; HF, heart failure; sK, serum potassium.
